# Probable Causes and Risk Factors for Positive SARS-CoV-2 Testing in Recovered Patients: Evidence From Guangzhou, China

**DOI:** 10.3389/fmed.2021.684101

**Published:** 2021-07-12

**Authors:** Lei Luo, Dan Liu, Zhoubin Zhang, Zhihao Li, Chaojun Xie, Zhenghe Wang, Zongqiu Chen, Peidong Zhang, Xiru Zhang, Yujie Zhang, Wenfang Zhong, Wenting Zhang, Pei Yang, Qingmei Huang, Weiqi Song, Hui Wang, Chen Mao

**Affiliations:** ^1^Guangzhou Center for Disease Control and Prevention, Guangzhou, China; ^2^Department of Epidemiology, School of Public Health, Southern Medical University, Guangzhou, China

**Keywords:** risk factors, COVID-19, discharged patients, cohort study, repositive

## Abstract

Some patients retested positive for SARS-CoV-2 following negative testing results and discharge. However, the potential risk factors associated with redetectable positive testing results in a large sample of patients who recovered from COVID-19 have not been well-estimated. A total of 745 discharged patients were enrolled between January 30, 2020, and September 9, 2020, in Guangzhou, China. Data on the clinical characteristics, comorbidities, drug therapy, RT-PCR testing, and contact modes to close contacts were collected. Patients who tested positive for SARS-CoV-2 after discharge were confirmed by guidelines issued by China. The repositive rate in different settings was calculated. Among 745 discharged patients, 157 (21.1%; 95% CI, 18.2–24.0%) tested repositive and the repositive rate was 16.8% (95% CI, 14.1–24.0%) for nasopharyngeal swabs and 9.7% (95% CI, 7.0–12.5%) for anal swabs. Among them, 55 (35.0%) were asymptomatic, 15 (9.6%) had mild symptoms, 83 (52.9%) had moderate symptoms, and 4 (2.6%) had severe symptoms at the first admission. The days from discharge to repositivity was 8.0 (IQR, 8.0–14.0). Most repositive patients were without clinical symptoms, and lymphocyte cell counts were higher than before being discharged. The likelihood of repositive testing for SARS-CoV-2 RNA was significantly higher among patients who were of younger age (OR, 3.88; 95% CI, 1.74–8.66, 0–17 years old), had asymptomatic severity (OR, 4.36; 95% CI, 1.47–12.95), and did not have clinical symptoms (OR, 1.89; 95% CI, 1.32–2.70, without fever). No other positive patients emerged within the families or close contacts of repositive patients. Our findings support prolonged but intermittent viral shedding as the probable cause for this phenomenon; we need to familiarize with the possibility that the virus will remain endemic.

## Introduction

Since the outbreak in December 2019, coronavirus disease 2019 (COVID-19), caused by severe acute respiratory syndrome coronavirus 2 (SARS-CoV-2), has given rise to a worldwide pandemic (1). As of April 4, 2021, 145 million COVID-19 patients and 3.1 million deaths have been reported globally (2). At the same time, tens of millions of patients with COVID-19 have recovered and been discharged from the hospital. However, some patients affected by COVID-19 who fully met the criteria for discontinuation of quarantine and subsequently report positive real-time reverse transcriptase-polymerase chain reaction (RT-PCR) again (hereafter referred as “repositive”) at a follow-up visit (3–8), which increases the complexity of disease control and has attracted widespread concern.

Several studies, mainly case reports, have been performed to investigate the clinical characteristics and virologic course of discharged patients (3–8). However, to date, many questions about repositive patients have not been answered; these questions include the overall prognosis of patients with COVID-19 after meeting the criteria for hospital discharge, the potential risk factors associated with redetectable positive test results, and whether the persistent presence of virus fragments means that the discharged patient is still contagious. As the number of discharged patients increases, effective management becomes critical to successfully reducing the spread of SARS-CoV-2. To promote the comprehensive rehabilitation of COVID-19 patients, China has implemented a series of measures for discharged COVID-19 patients, including management of quarantine, regular follow-up, health monitoring, and rehabilitation therapy, which provide empirical information and evidence support for the management of patients with COVID-19 (9).

Up till September 9, 2020, Guangzhou, China, recorded a total of 745 COVID-19 patients, of whom all have been discharged. We conducted a retrospective cohort study of all discharged patients to examine those who are repositive, describe their clinical and epidemiological outcomes, and analyze the predictors of repositive status.

## Materials and Methods

### Patients and Study Design

Between January 30, 2020, and September 9, 2020, a total of 745 patients who officially recovered from COVID-19 were discharged from the hospital and enrolled in this study in Guangzhou, China. Only COVID-19 patients who met all the following criteria of China (10) could be discharged from the hospital: (1) body temperature returns to normal for more than three consecutive days; (2) significant improvement in any symptoms, such as fever, dry cough, and expectoration; (3) substantial improvement in acute exudative lesions on chest computed tomography (CT) images; and (4) negative RT-PCR testing for SARS-CoV-2 RNA of nasopharyngeal swabs, anal swabs, and other respiratory specimens for two consecutive times (at least 24 h apart).

**Figure 1:**
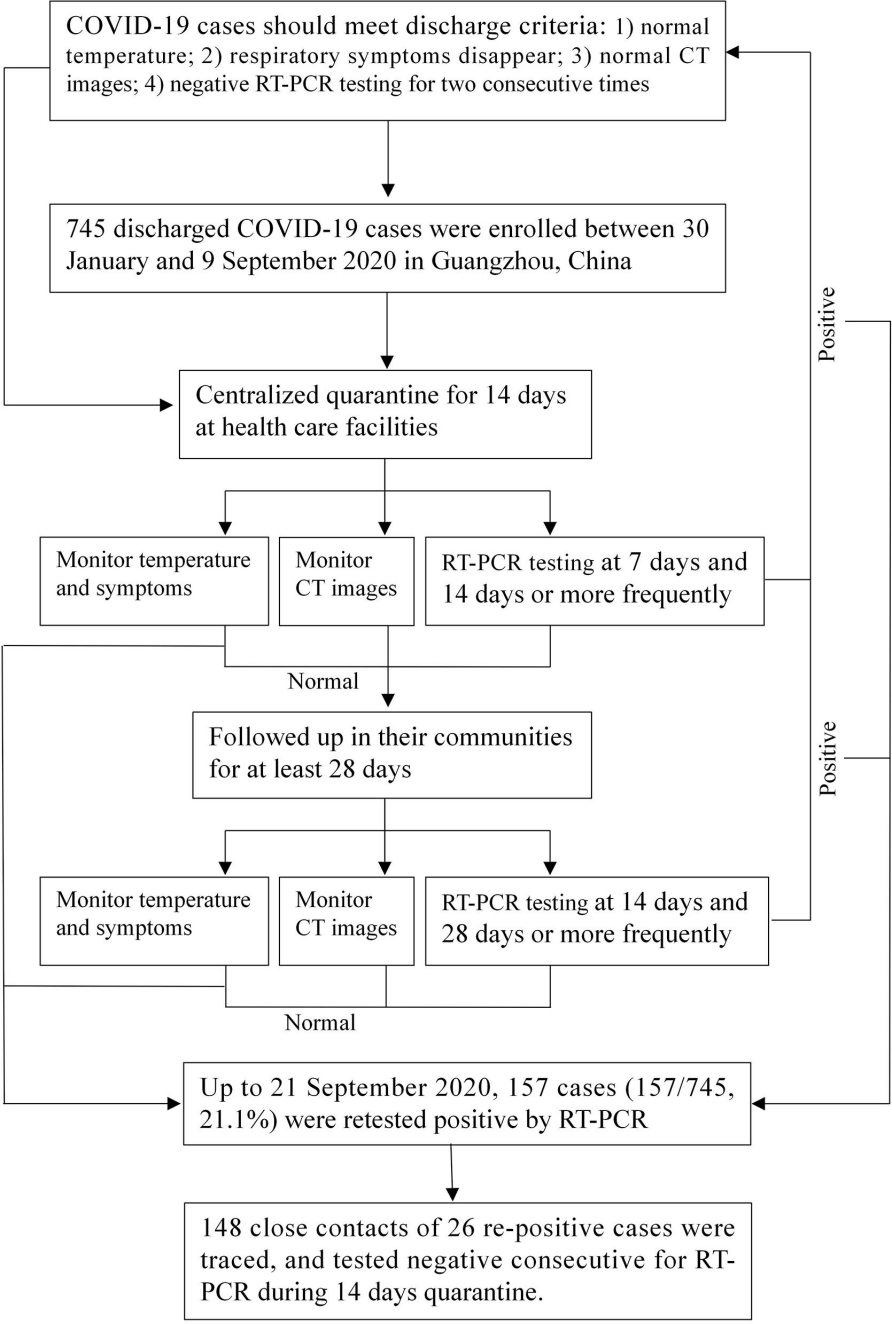
Study flow diagram. CT image, computed tomography image.

All discharged patients were required to undergo 14 days of quarantine in designated healthcare facilities and 28 days of community follow-up to observe their clinical symptoms and RT-PCR testing results. The repositive patients were re-admitted to the hospital for therapy and their close contacts were traced. The remaining discharged patients who continued to have negative RT-PCR testing results were closely followed up in their communities. Figure 1 shows the flowchart of the management of discharged patients.

These data were collected as part of a continuing public health response required by the National Health Commission of China and hence was determined not to be human subjects research and therefore was considered exempt from institutional review board approval after consultation with GZCDC. Patients were informed about the surveillance before providing written consent, and data were collected and anonymized for analysis. All analyses of personally identifiable data took place onsite at the GZCDC.

### Definition of Repositive Patients

Based on open reading frame 1ab (ORF 1ab) and nucleocapsid (N) protein genes in the SARS-CoV-2 genome, RT-PCR was performed to assess the results (11, 12). If the cycle threshold (Ct) value of RT-PCR is <37, the sample is positive; if the Ct value ranges between 37 and 40, and if the amplification curve has an obvious peak, then the sample is considered positive. Otherwise, the sample is considered as negative.

The following three conditions are considered positive. First, the two targets, ORF 1ab and N protein, are both positive. Second, in case of the result showing positivity for one target, samples shall be recollected for another test. If it is still positive for a single target, the result should be considered positive. Third, if two types of specimens show one single target as positive at the same time or if one target is positive in two samples of the same type, then the result should be considered positive.

### Management for Discharged Patients

After discharge from the hospital, patients were put under centralized quarantine and health monitoring for 14 days at designated healthcare facilities. During the quarantine period, the discharged patients lived in a well-ventilated single room, dined separately, and practiced hand hygiene. For children 14 years and younger, household management with medical observation can be used under the guidance of community health workers and family members can be with the child using personal protection and maintaining interpersonal distance. People with underlying medical conditions and elderly individuals cannot be with children who are discharged patients. Nasopharyngeal and anal specimens collected on the 1st, 7th, and 14th days or more frequently were sent to the laboratory for RT-PCR testing. The discharged patients were recorded for body temperature and respiratory or digestive tract symptoms (such as fever, dry cough, and diarrhea) every day. If the RT-PCR testing result was consecutively negative and no symptoms or CT images progressed (one or more times without fixed scanning time), these patients could return to normal life and be regularly followed up by the community. If they had a positive RT-PCR testing result, diagnosis and treatment should be conducted strictly in accordance with Chinese clinical guidance for COVID-19 (10).

After discharged patients completed 14 days of quarantine, it is recommended for these patients to be followed up in their communities for at least 28 days. GZCDC follows the health management plan for discharged patients released by the National Health Commission of China (9), and RT-PCR testing was performed on the 14th and 28th days after quarantine or more frequently.

### Close Contact Tracing of Repositive Patients

If the discharged patient was tested positive again, close contacts should be traced and followed up (13). The quarantine period should last until 14 days after the last contact without effective protection with a positive retest patient. Samples including nasopharyngeal and anal swabs were all collected for RT-PCR diagnosis. Monitoring and evaluating close contacts were documented in a previous study (14).

### Data Collection

The information collected for COVID-19 patients included demographic characteristics (age, sex, and continent), comorbidities (hypertension, diabetes, etc.), drug therapy, severity, clinical symptoms (fever, dry cough, myalgia, etc.), radiological examinations (CT), and blood examinations (white blood cell count, lymphocyte cell count, and lymphocyte cell percentage) at the first admission. The second admission information of positive retest patients was also collected. The information collected for close contacts included demographic characteristics, quarantine site (healthcare facilities and home), frequency of contact (often, moderate, and occasional), and contact modes (household, public transportation, healthcare settings, workplaces, and entertainment places).

COVID-19 severity includes five categories (10): asymptomatic, mild, moderate, severe, and critical. Asymptomatic infected persons were those with etiological detection of SARA-CoV-2 in respiratory specimens or specific IgM detected in serum. Mild cases were those who had mild symptoms and no sign of pneumonia on chest imaging. Moderate cases are those who had fever and respiratory symptoms and signs of pneumonia. Severe cases were those who meet any of the falling criteria: (1) shortness of breath, RR ≥30 times/min; (2) oxygen saturation ≤93% at rest; and (3) alveolar oxygen partial pressure/fraction of inspiration O_2_ (PaO_2_/FiO_2_) ≤300 mmHg. Critical cases are those who meet any of the falling conditions: (1) respiratory failure requiring mechanical ventilation; (2) shock; and (3) patients combined with other organ failure needed ICU monitoring and treatment. In this study, we combined severe and critical cases into the severe group.

### Statistical Analysis

The repositive rate of SARS-CoV-2 was estimated by dividing the number of repositive patients by the number of COVID-19 patients. Categorical variables are described as absolute numbers and percentages (%). Skewed and normally distributed continuous variables are described as the median [interquartile range (IQR) or range] and mean [standard deviation (SD)], respectively. Chi-square tests and *t*-tests were used to compare characteristics between repositive patients or not.

Univariate and multivariable logistic regression models (15) were performed to estimate odds ratios (ORs) and 95% confidence intervals (95% CIs) for predictors of repositive status. Age (0–17, 18–44, 45–59, or ≥60 years), sex (male or female), continent (Asia, Africa, or others), severity (asymptomatic, mild, moderate, or severe), and clinical symptoms (fever, dry cough, expectoration, myalgia, diarrhea, or shortness of breath) at the first admission were included in the multivariable model.

Analyses were all performed with SAS software (version 9.4 for Windows, SAS Institute, Inc., Cary, NC, USA). Statistical tests were two-sided, and *p*-values of <0.05 were considered to indicate statistical significance.

## Results

### Clinical Characteristics of Repositive Patients

Among 745 patients, 157 of them (21.1%; 95% CI, 18.2–24.0) retested positive by RT-PCR. The re-positive rate by types of specimen was 16.8% (95% CI, 14.1–24.0%) for nasopharyngeal swabs and 9.7% (95% CI, 7.0–12.5%) for anal swabs. The re-positive rate by days from discharge was 0.1% (95% CI, 0.0–0.4%) for 1 day, 5.0% (95% CI, 3.4–6.5%) for 2–7 days, 11.3% (95% CI, 9.0–13.6%) for 8–14 days, and 4.7% (95% CI, 3.2–6.2%) for >14 days (Table 1). The re-positive rate at different stages of the epidemic of COVID-19 was 17.0% (13.1–20.9%) for domestic case stage, 22.4% (14.5–30.3%) for imported case stage, and 25.8% (20.7–30.9%) for imported case associated local epidemic stage (Supplementary Figure 1). The characteristics at the first admission of the 157 repositive patients are shown in Table 1. Repositive results were observed in patients in all age groups (age ranging from 3 months to 82.0 years, with a median age of 33.0 years), which was significantly younger than that of negative retest patients (median age of 38.0 years) (*p* = 0.001). The days from first admission to discharge of repositive patients were significantly shorter than those of negative retest patients (11.2 vs. 13.0 days). One in three (35.0%) repositive patients were asymptomatic compared with one in six (17.7%) negative retest patients (*p* < 0.001). The repositive patients had fewer comorbidities [such as cardiovascular disease, 1 (0.6%) vs. 29 (4.9%)] and were less likely to be treated with anti-infective drugs [58 (36.9%) vs. 308 (52.4%)] and in the ICU [4 (2.6%) vs. 37 (6.3%)]. Sampling and RT-PCR testing were performed with median number of 4.0 times (IQR, 3.0–6.0 times) for repositive patients, which was significantly greater than that of negative retest patients (*p* < 0.001). The days for discharged patients retested positive was 8.0 (IQR, 8.0–14.0 days) (Table 1 and Supplementary Figure 2).

**Table 1:** Characteristic of 157 repositive and 588 non-repositive patients with COVID-19.

**Characteristic**	**All patients (*n* = 745)**	**Non-repositive (*n* = 588)**	**Repositive (*n* = 157)**	***P*-value**
Total repositive rate, % (95% CI)	–	–	21.1 (18.2–24.0)	–
**Repositive rate by type of specimen**				
From nasopharyngeal swab (*n* = 119)	–	–	16.8 (14.1–24.0)	–
From anal swab (*n* = 43)	–	–	9.7 (7.0–12.5)	–
**Repositive rate by days from discharge**				
1 day (*n* = 1)	–	–	0.1 (0.0–0.4)	–
2–7 days (*n* = 37)	–	–	5.0 (3.4–6.5)	–
8–14 days (*n* = 84)	–	–	11.3 (9.0–13.6)	–
>14 days (*n* = 35)			4.7 (3.2–6.2)	
Median age, years (range)	37.0 (0.25, 90.0)	38.0 (0.33, 90.0)	33.0 (0.25, 82.0)	0.001
**Gender**, ***n*** **(%)**				0.080
Male	424 (56.9)	325 (55.3)	99 (63.1)	
Female	321 (43.1)	263 (44.7)	58 (36.9)	
**Severity**, ***n*** (%)^*^				<0.001
Asymptomatic	159 (21.3)	104 (17.7)	55 (35.0)	
Mild	81 (10.9)	66 (11.2)	15 (9.6)	
Moderate	468 (62.8)	385 (65.5)	83 (52.9)	
Severe	37 (5.0)	33 (5.6)	4 (2.6)	
**Comorbidities**, ***n*** **(%)**				
Hypertension	77 (10.3)	65 (11.1)	12 (7.6)	0.212
Diabetes	32 (4.3)	29 (4.9)	3 (1.9)	0.097
Lung diseases	22 (3.0)	18 (3.1)	4 (2.6)	0.736
Cardiovascular disease	30 (4.0)	29 (4.9)	1 (0.6)	0.015
Other chronic diseases	59 (7.9)	55 (9.4)	4 (2.6)	0.005
**Drug therapy**, ***n*** **(%)**				
Anti–infectious drugs	366 (49.1)	308 (52.4)	58 (36.9)	0.001
Hormone therapy drugs	22 (3.0)	21 (3.6)	1 (0.6)	0.054
Antivirals	38 (5.1)	29 (4.9)	9 (5.7)	0.686
Chloroquine phosphate	10 (1.3)	7 (1.2)	3 (1.9)	0.486
Traditional Chinese medicine	96 (12.9)	76 (12.9)	20 (12.7)	0.951
**ICU**, ***n*** **(%)**				0.068
No	704 (94.5)	551 (93.7)	153 (97.5)	
Yes	41 (5.5)	37 (6.3)	4 (2.6)	
**Median days, days (IQR)**				
From onset to admission	2.8 (1.0, 6.0)	2.0 (1.0, 6.0)	3.7 (1.8, 6.4)	0.065
From admission to discharge	12.2 (7.0, 20.0)	13.0 (8.0, 20.0)	11.2 (5.5, 16.4)	0.005
From discharge to repositivity	–	–	8.0 (8.0, 14.0)	–
**Median no. of RT–PCR testing**, ***n*** **(IQR)**				
Nasopharyngeal swab	2.0 (2.0, 2.0)	2.0 (2.0, 2.0)	3.0 (2.0, 4.0)	<0.001
Anal swab	2.0 (0.0, 2.0)	2.0 (0.0, 2.0)	2.0 (0.0, 3.0)	0.000
Any of above	4.0 (2.0, 4.0)	4.0 (2.0, 4.0)	4.0 (3.0, 6.0)	<0.001

* *All patients were updated by progression of illness at their first admission, and the most severe condition was their final severity designation*.

Characteristics of 157 repositive cases at first and second admission are shown in Table 2. At second admission, 4 (2.6%) patients had dry cough, 10 (6.4%) had expectoration and 2 (1.3%) had sore throat, which was lower than that of the first admission. No one presented gastrointestinal symptoms at second admission. Among the 127 patients who underwent CT examination, 104 (81.3%) patients had abnormal but obvious absorption. Lymphocyte cell counts and lymphocyte cell percentages were increased compared with those before (*p* < 0.001).

**Table 2:** Characteristic of 157 repositive patients at first and second admission.

**Characteristic**	**First admission**	**Second admission**	***P*-value**
**Clinical symptoms**, ***n*** **(%)**			
Fever	65 (41.4)	0 (0.0)	–
Dry cough	61 (38.9)	4 (2.6)	<0.001
Expectoration	25 (15.9)	10 (6.4)	0.004
Sore throat	28 (17.8)	2 (1.3)	<0.001
Fatigue	19 (12.1)	2 (1.3)	<0.001
Headache	17 (10.8)	0 (0.0)	–
Chill	5 (3.2)	0 (0.0)	–
Myalgia	12 (7.6)	0 (0.0)	–
Diarrhea	6 (3.8)	0 (0.0)	–
Shortness of breath	9 (5.7)	0 (0.0)	–
CT lung abnormalities, *n* (%)^*^	96 (82.1)	104 (81.3)	0.248
**Median blood biochemical index (IQR)^†^**			
WBC (10^9^/L)	5.6 (4.5, 6.7)	5.5 (4.8, 6.6)	0.430
Ly (10^9^/L)	1.4 (1.1, 2.0)	2.0 (1.6, 2.3)	<0.001
Ly%	29.2 (22.0, 38.0)	32.5 (28.7, 41.8)	0.014

*CT, computed tomography; WBC, white blood cell count; Ly, lymphocyte cell count; Ly%, lymphocyte cell percentage*.

* *Missing values: 40 at first admission, 29 at second admission*.

† *Missing values: 46 of WBC, 50 of lymphocyte, 57 of lymphocyte percentage at first admission; 32 of the three at second admission*.

### Risk Factors Associated With Repositive Status

We compared repositive rates in Table 3. A higher repositivity rate of males than females [23.4% (95% CI, 19.3–27.4) vs. 18.1% (13.9–22.3)] was observed, but this difference was not statistically significant. The highest repositive rate was observed in patients aged 0–17 years old (42.9%; 95% CI, 27.9–57.8%) with OR of 3.88 (95% CI, 1.74–8.66) compared with patients aged 60 years or over (*p* for trend = 0.0023). The asymptomatic patients also had a higher repositive rate (34.6%; 95% CI, 27.2–42.0%) with OR of 4.36 (95% CI, 1.47–12.95) compared with severe patients. In addition, the repositive cases were found in younger age groups among four severity of disease (Supplementary Table 1). Patients without symptoms, such as without fever (OR = 1.89; 95% CI, 1.32–2.70), was associated with an increased risk of repositivity (Table 3). In addition, comorbidities, CT lung abnormalities, and some clinical symptoms (such as fatigue, chills, and sore throat) were not separately assessed due to multicollinearity with age, severity, and other clinical symptoms, and the repositive rate of COVID-19 by these variables is shown in Supplementary Table 2.

**Table 3:** Risk factors associated with re-positivity among COVID-19 patients (*n* = 157).

**Characteristic**	**Repositive patients (*n*)**	**Repositive rate % (95% CI)**	**Crude odds ratio (95% CI)**	**Adjusted odds ratio (95% CI)**
Age group^*^				
0–17 years	18	42.9 (27.9–57.8)	3.88 (1.74–8.66)	2.58 (1.05–6.32)
18–44 years	95	22.0 (18.1–25.9)	1.46 (0.83–2.57)	0.96 (0.50–1.81)
45–59 years	27	16.3 (10.7–21.9)	1.01 (0.52–1.95)	0.79 (0.40–1.57)
≥60 years	17	16.2 (9.1–23.2)	1.00 (ref)	1.00 (ref)
**Sex**				
Male	99	23.4 (19.3–27.4)	1.38 (0.96–1.99)	1.45 (0.98–2.15)
Female	58	18.1 (13.9–22.3)	1.00 (ref)	1.00 (ref)
**Continent**				
Asia	125	20.0 (16.9–23.1)	1.00 (ref)	1.00 (ref)
Africa	29	27.6 (19.1–36.2)	1.53 (0.95–2.44)	0.73 (0.39–1.35)
Others	3	20.0 (0.0–40.2)	1.00 (0.28–3.60)	0.60 (0.15–2.37)
**Severity^†^^‡^**				
Asymptomatic	55	34.6 (27.2–42.0)	4.36 (1.47–12.95)	3.83 (1.07–13.71)
Mild	15	18.5 (10.1–27.0)	1.88 (0.58–6.10)	1.27 (0.34–4.72)
Moderate	83	17.7 (14.3–21.2)	1.78 (0.61–5.16)	1.63 (0.52–5.11)
Severe or critical	4	10.8 (0.8–20.8)	1.00 (ref)	1.00 (ref)
**Clinical symptoms**				
Fever				
No	92	26.7 (22.1–31.4)	1.89 (1.32–2.70)	1.51 (1.00–2.28)
Yes	65	16.2 (12.6–19.8)	1.00 (ref)	1.00 (ref)
Dry cough				
No	96	23.7 (19.6–27.9)	1.42 (0.99–2.04)	0.90 (0.57–1.42)
Yes	61	17.9 (13.9–22.0)	1.00 (ref)	1.00 (ref)
Expectoration				
No	132	22.4 (19.0–25.8)	1.51 (0.95–2.42)	1.05 (0.61–1.81)
Yes	25	16.0 (10.3–21.8)	1.00 (ref)	1.00 (ref)
Myalgia				
No	145	22.0 (18.8–25.2)	1.74 (0.92–3.29)	1.11 (0.56–2.20)
Yes	12	14.0 (6.6–21.3)	1.00 (ref)	1.00 (ref)
Diarrhea				
No	151	21.4 (18.4–24.5)	1.54 (0.64–3.74)	1.28 (0.50–3.26)
Yes	6	15.0 (3.9–26.1)	1.00 (ref)	1.00 (ref)
Shortness of breath				
No	148	21.7 (18.6–24.8)	1.63 (0.79–3.38)	0.91 (0.41–2.04)
Yes	9	14.5 (5.8–23.3)	1.00 (ref)	1.00 (ref)

* *p for trend = 0.002*.

† *All patients were updated by progression of illness at their first admission, and the most severe condition was their final severity designation*.

‡ *p for trend <0.001*.

We performed strata analysis according to the types of specimen, and the repositive rate of nasopharyngeal and anal swabs is shown in Supplementary Table 3. Generally, the overall repositive rate of anal swabs was lower than that of nasopharyngeal swabs except for the group of 0–17 years old [39.3% (95% CI, 21.2–57.4%) vs. 22.6% (95% CI, 7.9–37.3%)], mild severity [13.6% (95% CI, 3.5–23.8%) vs. 12.0% (95% CI, 4.7–19.4%)], and symptoms of diarrhea [10.0% (95% CI, 0.0–20.7%) vs. 8.1% (95% CI, 0.0–16.9%)].

### Infection in Close Contacts of Repositive Patients

Because all the discharged patients were put under centralized quarantine for 14 days at healthcare facilities, only 26 positive retest patients had close contacts, and 148 close contacts were traced. Table 4 presents the characteristics of repositive patients and close contacts. A total of 137 (92.6%) close contacts were quarantined at healthcare facilities, and 11 (7.4%) close contacts quarantined at home. After quarantine for 12.0 days (IQR, 6.0–14.0) at a healthcare facility or at home and 4.5 times (IQR, 3.0–10.0) of RT-PCR testing, 148 close contacts tested negative for SARS-CoV-2 RNA, and no suspicious clinical symptoms were reported.

**Table 4:** Characteristic of repositive patients and the close contacts.

**Characteristic**	**Repositive patients (*n* = 26)**	**Close contacts (*n* = 148)**
**Age group**, ***n*** **(%)**		
0–17 years	3 (11.5)	15 (10.1)
18–44 years	13 (50.0)	92 (62.2)
45–59 years	4 (15.4)	34 (23.0)
≥60 years	6 (23.1)	7 (4.7)
**Sex**, ***n*** **(%)**		
Male	17 (65.4)	89 (60.1)
Female	9 (34.6)	59 (39.9)
**Quarantine site**, ***n*** **(%)**		
Healthcare facilities	26 (100.0)	137 (92.6)
Home	0 (0.0)	11 (7.4)
**Frequency of contact**, ***n*** **(%)**		
Often	11 (26.8)	27 (18.2)
Moderate	10 (24.4)	24 (16.2)
Occasional	20 (48.8)	97 (65.5)
**Contact modes**, ***n*** **(%)**		
Household	14 (29.8)	42 (28.4)
Public transportation	18 (38.3)	61 (41.2)
Healthcare settings	2 (4.3)	2 (1.4)
Workplaces	6 (12.8)	12 (8.1)
Entertainment places	7 (14.9)	31 (21.0)
**Severity of patients**, ***n*** (%)^*^		
Asymptomatic	3 (11.5)	40 (27.0)
Mild	3 (11.5)	8 (5.4)
Moderate	18 (69.2)	96 (64.9)
Severe	2 (7.7)	4 (2.7)
**Days from discharge to repositivity**		
3–13	18	–
≥14	8	–
Median days of quarantine, days (IQR)	–	12.0 (6.0, 14.0)
Median no. of RT-PCR testing, *n* (IQR)	–	4.5 (3.0, 10.0)
Infected close contacts, *n* (%)	–	0 (0.0)

* *Severity of patients means that the progression of illness at first admission of repositive patients, and the severity of close contacts is not the symptoms of close contacts, but the symptoms of patients who have contact the close contacts*.

## Discussion

We found that the repositive rate of SARS-CoV-2 was 21.1% among discharged patients at a follow-up visit after at least 6 weeks. They reported positive RT-PCR testing results with 8.0 days after discharge. Over 4 in 10 children were found to be positive again; in contrast, the repositive rate of SARS-CoV-2 in middle-aged and elderly individuals was 16%. Moreover, patients with more clinically severe disease were less likely to have repositive testing results than those who were asymptomatic. Manifestation of certain symptoms at first admission, such as fever, was also associated with a lower risk for repositivity. Based on the Chinese guidelines for discharged patients (9), repositive patients were required to quarantine for a second time. No other positive patients emerged within their families and close contacts.

Several studies have been performed to investigate the percentage of repositivity of discharged patients (3, 16, 17). Previous studies reported that the repositive rate ranged from 6.9% to 69.0% for discharged patients (16–19). However, the studies were limited to a small number of patients with mild or moderate infection. In our study, we evaluated the overall prognosis of patients with COVID-19 after meeting the criteria for discharge in Guangzhou, China. Our study has lasted more than 7 months since the start of the outbreak, which was far longer than other studies (most lasted for 1 or 2 months) (7, 17, 20, 21) and to some extent represented the overall prognosis of the disease. After screening 745 discharged patients, the repositive rate was over 20% (157/745), which was higher than that in other countries, such as Brunei Darussalam (21/106, 19.8%) (19) and Italy (22/131, 16.7%) (18), and this may be due to the longer follow-up time, more stringent monitoring, and higher frequency of RT-PCR testing in China. In our study, one patient tested repositive on the first day (Table 1), which may be attributed to the false negative of last time. The repositive rate at different stages of the epidemic of COVID-19 was increased (Supplementary Figure 1), which may be related to improvements of testing reagents and changes of discharge standards (22, 23).

Some reports suggested reinfection as a possible cause (24); our findings do not support this. According to the Chinese clinical guidance for COVID-19 (10), all repositive patients should test negative for nasopharyngeal and anal swabs for two successive tests before discharge. Then, all discharged patients were continuously quarantined in designated healthcare facilities with strict interventions on disease transmission. Thus, the identification of another positive SARS-CoV-2 test during the quarantine period likely excludes the possibility that positive retest patients are caused by secondary viral infection. A recent study also experimentally confirmed that the virus was not a secondary infection (8).

Abnormal CT and lymphopenia are common and correlate with poor clinical outcomes in patients with COVID-19 (25). In our study, most positive retest patients at the second admission showed increased lymphocyte cell counts, and CT examination showed abnormal but obvious improvements, suggesting that repositive patients have no obvious disease progression and reactivation is also unlikely. In addition, current evidence to date showed that the probable causes of repositivity with false-negative or false-positive results of qPCR are the most frequent. However, in our study, sampling and RT-PCR testing were performed with 4.0 times for all discharged patients, and samples including nasopharyngeal and anal swabs were all collected for RT-PCR diagnosis in an attempt to reduce the chance of false negatives caused by differences in primer specificity and sensitivity.

At present, virological studies have reported prolonged viral shedding in SARS-CoV-2-positive patients, which took 2 to 3 weeks or longer (26–30). Genetic studies on SARS suggested that host responses might result in undetectable levels of nasopharyngeal virus shedding at certain times (31). Our findings support prolonged but intermittent viral shedding as the most plausible explanation. In our study, the days of first hospitalization were shorter in repositive patients than in negative retest patients, and the observation of repositive patients was not random and was mainly observed in young patients without severe clinical symptoms, suggesting that the SARS-CoV-2 virus may not be completely eliminated due to the lighter symptoms and the faster attainment of the discharge standard.

Whether discharged patients have infectivity is an issue of concern around the world at present. However, positive testing induced by viral RNA shedding of SARS-CoV-2 may not necessarily imply an ability to transmit infection, unless there is proof that the virus can be isolated and cultured from the particular samples. While we did not culture the samples in our study, other studies reported that no infectious strain could be obtained by culture, and no full-length viral genomes could be sequenced using samples of positive retest patients (20). Among positive retest patients in our study, no families or close contacts of positive retest patients tested positive, which was consistent with current studies (20, 32).

## Limitations

Our study has some limitations. First, as our data were based on the public health response to COVID-19, sample collection did not follow a stringent study design. Therefore, some of the patients, especially in the early stage, had missing fecal samples. However, patients who retest positive from anal swab are not recommended to follow-up since no evidence of fecal–oral transmission have been described for SARS-CoV2 so far (33, 34). Second, nasopharyngeal swab samples cannot differentiate whether the virus comes from the nasopharynx or from secretions from the lower respiratory tract; thus, virus elimination in the lower respiratory tract cannot be confirmed. In contrast, the positive rate of RT-PCR testing through alveolar lavage fluid may be higher. However, this method is invasive and cannot be widely performed in clinical practice. In our opinion, both qualities of respiratory samples and the variability of technique sensitivity can be attributed to the influencing factors of repositivity. Third, as the discharge patients were usually placed under centralized quarantine and medical observation, the infectivity of the positive retest patients might be underestimated. Fourth, we could not provide the serological status in terms of IgG against the Spike protein and/or N protein in these discharged patients during the follow-up and we did not perform any infectivity test *in vitro* to validate the contagiosity of repositive patients.

## Conclusions

We found that the repositive rate of discharged patients was higher (21.1%) than commonly reported. The observation of positive retest patients was not random and was mainly observed in young patients without severe clinical symptoms. No other positive patients emerged within the families or close contacts of patients who resulted “repositive.” Our findings support prolonged but intermittent viral shedding as the probable cause for this phenomenon; we need to familiarize with the possibility that the virus will remain endemic.

## Data Availability Statement

The raw data supporting the conclusions of this article will be made available by the authors, without undue reservation.

## Author Contributions

DL contributed to the statistical analyses and drafted the manuscript. CM, LL, DL, ZL, ZW, PZ, XZ, YZ, WZho, and WS revised the final manuscript. ZZ, CX, ZC, WZha, PY, and QH collected the epidemiological and clinical data. HW and DL are responsible for summarizing all epidemiological and clinical data, and contributed to data cleaning. All authors critically reviewed the manuscript for important intellectual content.

## Conflict of Interest

The authors declare that the research was conducted in the absence of any commercial or financial relationships that could be construed as a potential conflict of interest.
