# Supplementary material for: Probable Causes and Risk Factors for Positive SARS-CoV-2 Testing in Recovered Patients: Evidence From Guangzhou, China
Source: Front Med (Lausanne). 2021 Jul 12;8:684101. doi: 10.3389/fmed.2021.684101 (PMC8311025; doi:10.3389/fmed.2021.684101)
Supplement: Supplementary file 1 [file Data_Sheet_1.docx]

**Table S1.**Repositive rate among 745 COVID-19 cases by severity of diseaseaccording to the age

| **Characteristic** | **Re-positive cases (n)** | **Re-positive incidence (%)** |
| --- | --- | --- |
| Asymptomatic (n=159) |  |  |
| 0-17 y | 6 | 46.2 |
| 18-44 y | 43 | 33.1 |
| 45-59 y | 6 | 37.5 |
| ≥60 y | 0 | 0 |
| Mild (n=81) |  |  |
| 0-17 y | 4 | 33.3 |
| 18-44 y | 9 | 14.5 |
| 45-59 y | 1 | 16.7 |
| ≥60 y | 1 | 100.0 |
| Moderate (n=468) |  |  |
| 0-17 y | 8 | 47.1 |
| 18-44 y | 41 | 17.5 |
| 45-59 y | 19 | 14.4 |
| ≥60 y | 15 | 17.7 |
| Severe or critical (n=37) |  |  |
| 0-17 y | 0 | 0 |
| 18-44 y | 2 | 33.3 |
| 45-59 y | 1 | 8.3 |
| ≥60 y | 1 | 5.3 |

**Table S2.**Repositive rate among 745 COVID-19 cases

| **Characteristic** | **Re-positive cases (n)** | **Re-positive incidence**  **% (95%CI)** | ***P* value** |
| --- | --- | --- | --- |
| **Clinical Symptoms** |  |  |  |
| Sore throat |  |  | 0.060 |
| No | 129 | 22.6 (19.2-26.1) |  |
| Yes | 28 | 16.0 (10.6-21.4) |  |
| Fatigue |  |  | 0.022 |
| No | 138 | 22.7 (19.4-26.0) |  |
| Yes | 19 | 13.9 (8.1-19.7) |  |
| Headache |  |  | 0.080 |
| No | 140 | 22.2 (19.0-25.4) |  |
| Yes | 17 | 14.9 (8.4-21.5) |  |
| Chill |  |  | <0.001 |
| No | 152 | 23.7 (20.4-27.0) |  |
| Yes | 5 | 4.9 (0.7-9.0) |  |
| **CT lung abnormalities** * |  |  | 0.050 |
| No | 21 | 25.0 (15.7-34.3) |  |
| Yes | 96 | 16.3 (13.3-19.3) |  |
| **Comorbidities** |  |  | 0.003 |
| No | 138 | 23.4 (19.9-26.8) |  |
| Yes | 19 | 12.3 (7.1-17.5) |  |

CT = computed tomography.

*Missing values: 73.

**Table S3.**Repositive rate among COVID-19 cases by nasopharyngeal and anal swabs

| **Characteristic** | Nasopharyngeal swabs (n=707) | | |  | Anal swabs (n=442) | | |
| --- | --- | --- | --- | --- | --- | --- | --- |
|  | Re-positive cases (n) | Re-positive incidence  % (95%CI) | *P* value |  | Re-positive cases (n) | Re-positive incidence  % (95%CI) | *P* value |
| **Age group** |  |  | 0.201 |  |  |  | <0.001 |
| 0-17 y | 7 | 22.6 (7.9-37.3) |  |  | 11 | 39.3 (21.2-57.4) |  |
| 18-44 y | 78 | 18.8 (15.0-22.6) |  |  | 20 | 9.1 (5.3-12.8) |  |
| 45-59 y | 20 | 12.6 (7.4-17.7) |  |  | 8 | 7.2 (2.4-12.0) |  |
| ≥60 y | 14 | 13.7 (7.1-20.4) |  |  | 4 | 4.9 (0.2-9.5) |  |
| **Sex** |  |  | 0.294 |  |  |  | 0.007 |
| Male | 72 | 18.1 (14.4-21.9) |  |  | 30 | 13.5 (9.0-18.0) |  |
| Female | 47 | 15.2 (11.2-19.2) |  |  | 13 | 5.9 (1.6-9.0) |  |
| **Continent** |  |  | 0.005 |  |  |  | 0.433 |
| Asian | 87 | 14.8 (12.0-17.7) |  |  | 43 | 10.1 (7.2-12.9) |  |
| Africa | 29 | 27.6 (19.1-36.2) |  |  | 0 | 0.0 (0.0-0.0) |  |
| Others | 3 | 20.0 (4.3-48.1) |  |  | 0 | 0.0 (0.0-0.0) |  |
| **Severity** † |  |  | <0.001 |  |  |  | 0.669 |
| Asymptomatic | 52 | 33.3 (25.9-40.7) |  |  | 3 | 8.8 (1.9-23.7) |  |
| Mild | 9 | 12.0 (4.7-19.4) |  |  | 6 | 13.6 (3.5-23.8) |  |
| Moderate | 55 | 12.5 (9.4-15.6) |  |  | 33 | 9.7 (6.5-12.8) |  |
| Severe or critical | 3 | 8.3 (1.8-22.5) |  |  | 1 | 4.4 (0.0-12.7) |  |
| **Clinical Symptoms** |  |  |  |  |  |  |  |
| Fever |  |  | <0.001 |  |  |  | 0.281 |
| No | 76 | 23.2 (18.6-27.7) |  |  | 19 | 11.7 (6.8-16.7) |  |
| Yes | 43 | 11.4 (8.2-14.5) |  |  | 24 | 8.6 (5.3-11.9) |  |
| Dry cough |  |  | 0.009 |  |  |  | 0.892 |
| No | 78 | 20.2 (16.2-24.2) |  |  | 19 | 10.0 (5.7-14.2) |  |
| Yes | 41 | 12.8 (9.2-16.5) |  |  | 24 | 9.6 (5.9-13.2) |  |
| Expectoration |  |  | 0.184 |  |  |  | 0.368 |
| No | 99 | 17.8 (14.6-21.0) |  |  | 34 | 10.5 (7.2-13.8) |  |
| Yes | 20 | 13.3 (7.8-18.7) |  |  | 9 | 7.6 (2.8-12.4) |  |
| Myalgia |  |  | 0.307 |  |  |  | 0.013 |
| No | 108 | 17.4 (14.4-20.3) |  |  | 42 | 11.2 (8.0-14.4) |  |
| Yes | 11 | 12.9 (5.8-20.1) |  |  | 1 | 1.5 (0.0-4.3) |  |
| Diarrhea |  |  | 0.145 |  |  |  | 0.959 |
| No | 116 | 17.3 (14.5-20.2) |  |  | 40 | 9.7 (6.9-12.6) |  |
| Yes | 3 | 8.1 (0.0-16.9) |  |  | 3 | 10.0 (0.0-20.7) |  |
| Shortness of breath |  |  | 0.153 |  |  |  | 0.465 |
| No | 113 | 17.4 (14.5-20.4) |  |  | 40 | 10.1 (7.1-13.0) |  |
| Yes | 6 | 10.2 (2.5-17.9) |  |  | 3 | 6.7 (0.0-14.0) |  |
| Sore throat |  |  | 0.034 |  |  |  | 0.319 |
| No | 100 | 18.5 (15.2-21.8) |  |  | 33 | 10.7 (7.2-14.1) |  |
| Yes | 19 | 11.5 (6.6-16.3) |  |  | 10 | 7.6 (3.1-12.1) |  |
| Fatigue |  |  | 0.034 |  |  |  | 0.135 |
| No | 105 | 18.3 (15.1-21.4) |  |  | 37 | 10.9 (7.6-14.2) |  |
| Yes | 14 | 10.6 (5.4-15.9) |  |  | 6 | 5.9 (1.3-10.5) |  |
| Headache |  |  | 0.126 |  |  |  | 0.206 |
| No | 106 | 17.8 (14.7-20.8) |  |  | 38 | 10.6 (7.4-13.8) |  |
| Yes | 13 | 11.8 (5.8-17.9) |  |  | 5 | 6.0 (0.9-11.1) |  |
| Chill |  |  | 0.000 |  |  |  | 0.002 |
| No | 115 | 19.0 (15.9-22.1) |  |  | 42 | 11.9 (8.5-15.2) |  |
| Yes | 4 | 3.9 (0.2-7.7) |  |  | 1 | 1.1 (0.0-3.4) |  |
| **CT lung abnormalities** * |  |  | 0.090 |  |  |  | 0.263 |
| No | 15 | 19.2 (10.5-28.0) |  |  | 6 | 14.0 (3.6-2.4) |  |
| Yes | 69 | 12.3 (9.6-15.0) |  |  | 32 | 8.7 (5.8-11.6) |  |
| **Comorbidities** |  |  | 0.003 |  |  |  | 0.062 |
| No | 106 | 19.0 (15.7-22.2) |  |  | 37 | 11.3 (7.9-14.7) |  |
| Yes | 13 | 8.8 (4.2-13.3) |  |  | 6 | 5.3 (1.2-9.4) |  |

CT = computed tomography.

* Missing values: 73.

† All patients were updated by progression of illness at their first admission, and the most severe condition was their final severity designation.


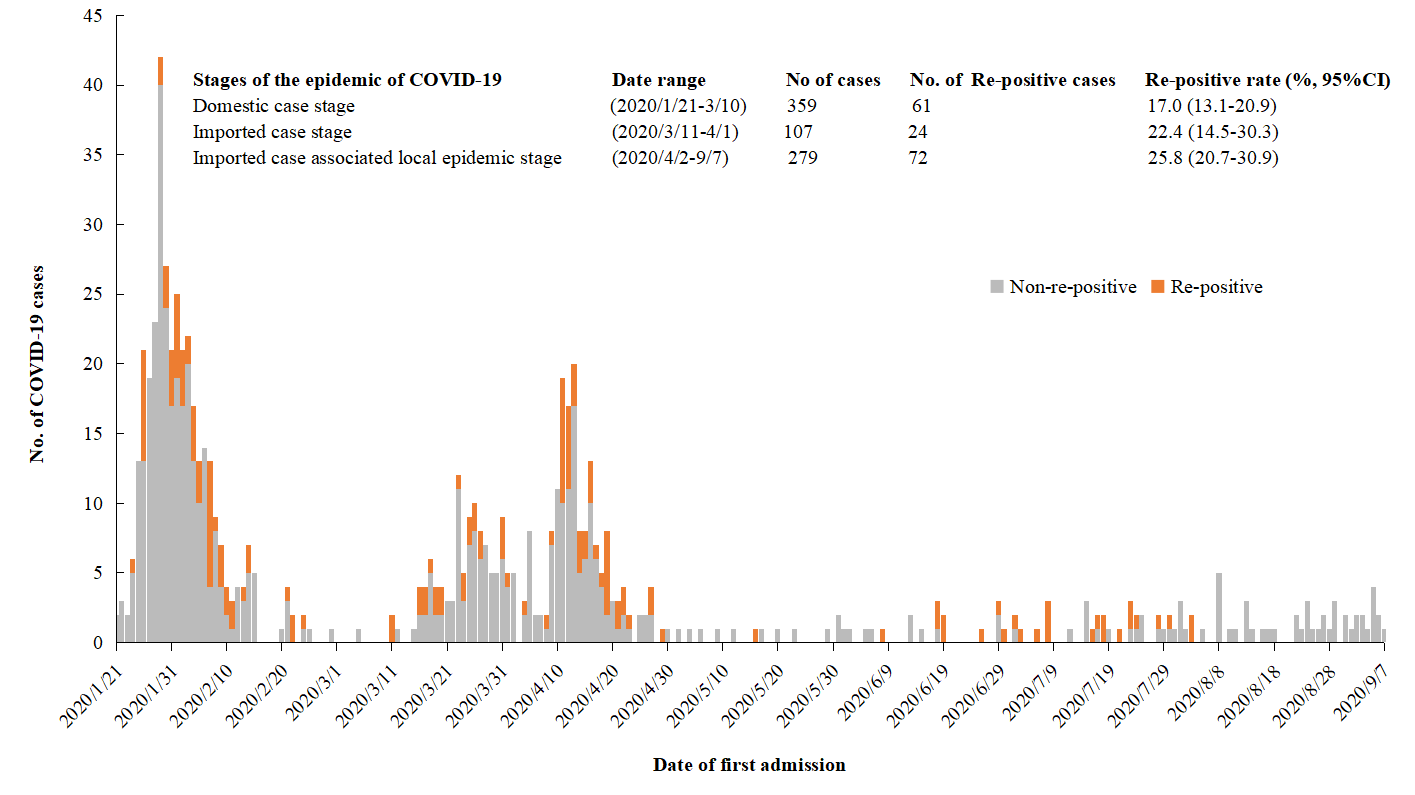


**Figure S1.** Distribution of repositive rate at different stages of the epidemic of COVID-19


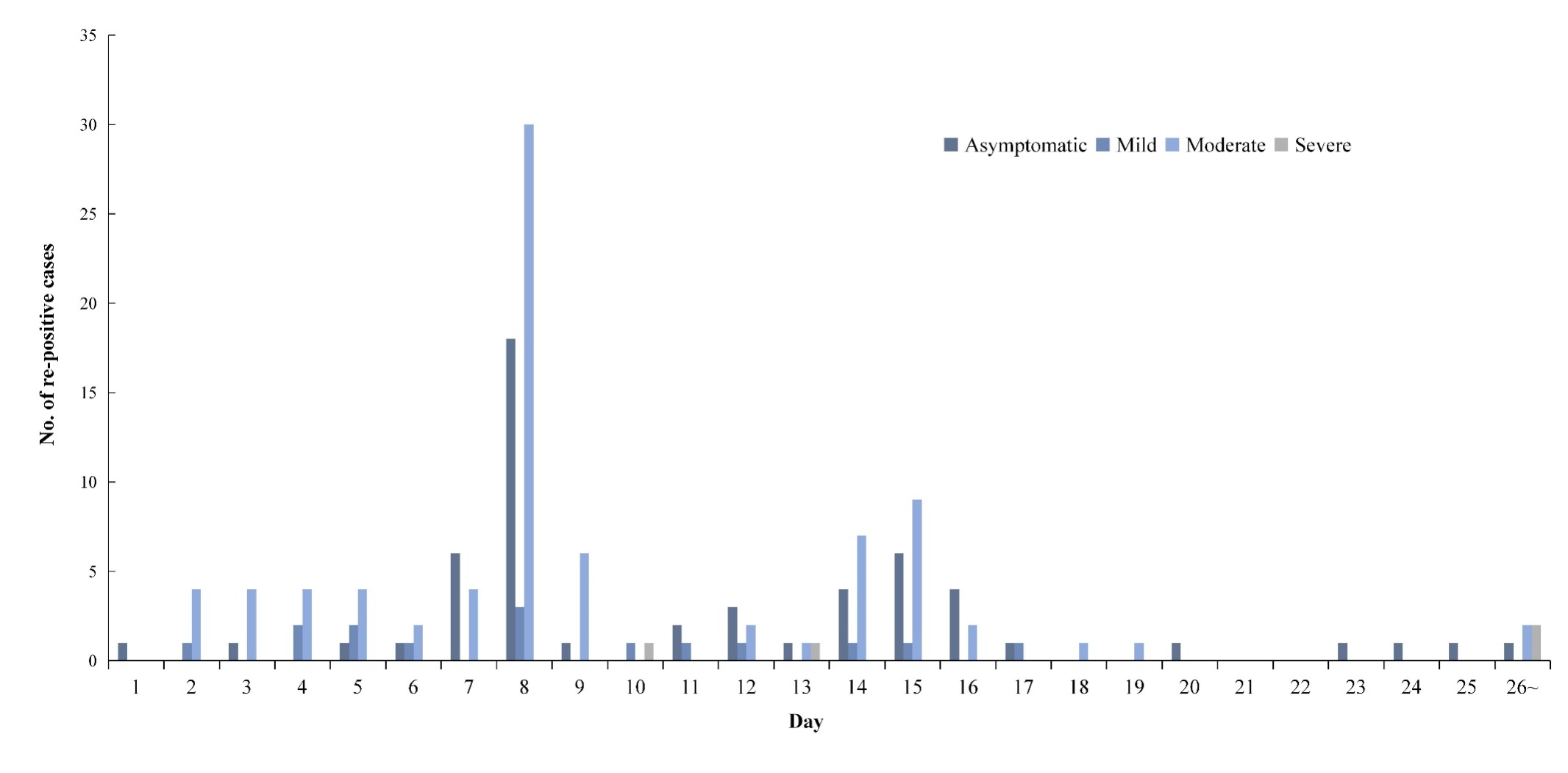


**Figure S2.**Distribution of days from discharge to repositivity among repositive patients according to the severity at first hospitalization
